# Targeting HNRNPU to overcome cisplatin resistance in bladder cancer

**DOI:** 10.1186/s12943-022-01517-9

**Published:** 2022-02-07

**Authors:** Zhen-duo Shi, Lin Hao, Xiao-xiao Han, Zhuo-Xun Wu, Kun Pang, Yang Dong, Jia-xin Qin, Guang-yue Wang, Xuan-ming Zhang, Tian Xia, Qing Liang, Yan Zhao, Rui Li, Shao-qi Zhang, Jun-hao Zhang, Jian-gang Chen, Gong-cheng Wang, Zhe-Sheng Chen, Cong-hui Han

**Affiliations:** 1 Department of Urology, Xuzhou Clinical School of Xuzhou Medical University, Jiangsu, China; 2 Department of Urology, Xuzhou Central Hospital, Xuzhou, Jiangsu China; 3 Clinical and Translational Research Center of Shanghai First Maternity and Infant Hospital, Shanghai Key Laboratory of Signaling and Disease Research, Frontier Science Center for Stem Cell Research, School of Life Sciences and Technology, Tongji University, Shanghai, China; 4 Department of Pharmaceutical Sciences, College of Pharmacy and Health Sciences, St. John’s University, Queens, NY 11439 USA; 5 Graduate School of Bengbu Medical College, Anhui, China; 6 Nanjing Drum Tower Hospital Group, Suqian Hospital, Jiangsu, China; 7 Affiliated Hospital of Guizhou Medical University, Guizhou, China; 8 Department of Urology, The Second Affiliated Hospital of Nantong University, Jiangsu, China; 9 Department of Urology, Huai’an First People’s Hospital, Nanjing Medical University, Jiangsu, China; 10 Department of Urology, Heilongjiang Provincial Hospital, Heilongjiang, China; 11 College of Life Sciences, Jiangsu Normal University, Jiangsu, China

**Keywords:** Genome-wide CRISPR screening, HNRNPU, Cisplatin, Bladder urothelial carcinoma

## Abstract

**Purpose:**

The overall response of cisplatin-based chemotherapy in bladder urothelial carcinoma (BUC) remains unsatisfactory due to the complex pathological subtypes, genomic difference, and drug resistance. The genes that associated with cisplatin resistance remain unclear. Herein, we aimed to identify the cisplatin resistance associated genes in BUC.

**Experimental design:**

The cytotoxicity of cisplatin was evaluated in six bladder cancer cell lines to compare their responses to cisplatin. The T24 cancer cells exhibited the lowest sensitivity to cisplatin and was therefore selected to explore the mechanisms of drug resistance. We performed genome-wide CRISPR screening in T24 cancer cells in vitro, and identified that the gene heterogeneous nuclear ribonucleoprotein U (HNRNPU) was the top candidate gene related to cisplatin resistance. Epigenetic and transcriptional profiles of HNRNPU-depleted cells after cisplatin treatment were analyzed to investigate the relationship between HNRNPU and cisplatin resistance. In vivo experiments were also performed to demonstrate the function of HNRNPU depletion in cisplatin sensitivity.

**Results:**

Significant correlation was found between HNRNPU expression level and sensitivity to cisplatin in bladder cancer cell lines. In the high HNRNPU expressing T24 cancer cells, knockout of HNRNPU inhibited cell proliferation, invasion, and migration. In addition, loss of HNRNPU promoted apoptosis and S-phase arrest in the T24 cells treated with cisplatin. Data from The Cancer Genome Atlas (TCGA) demonstrated that HNRNPU expression was significantly higher in tumor tissues than in normal tissues. High HNRNPU level was negatively correlated with patient survival. Transcriptomic profiling analysis showed that knockout of HNRNPU enhanced cisplatin sensitivity by regulating DNA damage repair genes. Furthermore, it was found that HNRNPU regulates chemosensitivity by affecting the expression of neurofibromin 1 (NF1).

**Conclusions:**

Our study demonstrated that HNRNPU expression is associated with cisplatin sensitivity in bladder urothelial carcinoma cells. Inhibition of HNRNPU could be a potential therapy for cisplatin-resistant bladder cancer.

**Supplementary Information:**

The online version contains supplementary material available at 10.1186/s12943-022-01517-9.

## Introduction

Bladder urothelial carcinoma (BUC) is the ninth most common cancer with high mortality worldwide [1]. Timely surgical treatment or radiotherapy has become the standard therapeutic approaches for patients with localized and non-muscle-invasive BUC. However, these treatments are often insufficient in recurrent or distant metastatic diseases, especially for muscle-invasive BUC, which usually forms micro-metastases [2]. Hence, systemic chemotherapy has been concurrently employed to control BUC and alleviate symptoms.

Cisplatin-based chemotherapy is the first-line treatment for most cancers, including bladder cancer, breast cancer, and colorectal cancer. Although this treatment achieved an ideal reduction in the risk of bladder cancer-induced death, the overall response rate is less than 50% in clinical settings [3]. It has been suggested that the complex histological subtypes, genomic effects, and acquired cisplatin resistance might attenuate its efficiency [4–6], and thus, there is substantial room to improve the therapeutic response. Baltazar et al. found that overexpression of CD147 and monocarboxylate transporter 1 might weaken cisplatin sensitivity in bladder cancer [7]. Other groups indicated that silencing of the pyruvate kinase M2 in vitro could overcome cisplatin resistance [8, 9]. Unfortunately, the molecules that associated with the efficacy of cisplatin remain unclear [10]. Therefore, there is an essential need to further identify the underlying mechanisms of cisplatin resistance.

Genome-wide analyses using the clustered regularly interspaced short palindrome repeat-associated nuclease Cas9 (CRISPR-Cas9) system is an emerging new tool with high efficiency and flexibility, which is an ideal choice for investigating gene functions [11, 12]. The CRISPR library has been reported to target thousands of genes and enable negative or positive alteration screening both in vivo and in vitro. Thus, it could be used to evaluate the specific relationship between genomic variants and drug response [13]. For example, Xu et al. demonstrated that ELP5 might be a candidate gene responsible for gemcitabine sensitivity in gallbladder cancer cells in the CRISPR screen [14]. James et al. found that loss of MSH2 promoted cisplatin resistance in BUC cells using CRISPR screening [15].

In order to understand the underlying principle of cisplatin sensitivity in BUC, we investigated genes associated with cisplatin response in T24 cells using CRISPR screen. The results showed that HNRNPU inhibition weakened the tolerance of cisplatin treatment by promoting cisplatin-mediated cell cycle arrest and apoptosis, in addition to reducing cell migration. Furthermore, we identified that the loss of HNRNPU regulated the interphase chromosome structure of multiple genes, which might be associated with the drug response in BUC.

## Materias and methods

### Cell culture

Human bladder cancer cells (T24, RT4, HT1197, SW780, RT112, HT1376) and human embryonic kidney HEK293T cells were obtained from ATCC. T24 and HEK293T were maintained in RPMI 1640 medium. The SW780, HT1197, HT1376, and RT112 cells were maintained in EMEM. RT4 cells were maintained in DMEM. All culture media were supplemented with 10% fetal bovine serum (Gibco), 100 mg/mL penicillin, and streptomycin solution (Thermo).

### Chemotherapeutic drug cytotoxicity assay

The bladder cancer cell lines were seeded in 96-well plates at 5,000 cells per well and incubated overnight to allow cell attachment. Different concentrations (0–100 μM) of cisplatin, paclitaxel, or doxorubicin were added to the designated wells and the plates were incubated for 3 days. After incubation, 10 µL CCK-8 solution was added to each well and incubated at 37 °C for 2 h, the light absorbance of each sample was then measured at 450 nm using a microplate reader (Biotek). The IC_50_ values were calculated using GraphPad Prism 8.1.

### CRISPR screening

The sgRNA library was obtained from Addgene. HEK 293 T cells were plated on 10-cm dishes to clone the lentiCRISPR vector and the control sgRNA (sgCtrl), following the protocol of GeCKO v2. Next, T24 cells were infected with slow virus at 0.4 functional multiplicity of infection (MOI) and selected by 2 µg/mL puromycin treatment for 48 h after transfection. Subsequently, 2 × 10^6^ transduced T24 cells were treated with 0.25 µM cisplatin or an equal volume of dimethylformamide (DMF) for 7 or 14 days. Finally, the cells were collected for subsequent genomic DNA analyses.

Genomic DNA was isolated using the TIANamp Genomic DNA Kit (Tiangen, China), and amplified by PCR, according to the manufacturer’s protocol. DNA fragments were selected with 2% agarose gels and subjected to HiSeq2500 (Illumina) for sequencing. The data were then analyzed using the MAGeCK software. The candidate genes in the DMF group were defined by a fold change < 2 or > -2 and Bayes facto *r* < 0. For the cisplatin-treated group, the gene was set at a fold change < -2 and Bayes factor > 2. The KEGG and BioCarta databases were used to determine the gene ontology (GO) and pathway analyses, respectively.

### High-content screening (HCS) analysis

To identify the effect of mixed sgRNAs, transfected T24 cells were seeded into 96-well plates at a density of 2000 cells per well, and then treated with 0.6 µM cisplatin or an equal volume of DMF for 5 days. To examine the function of single sgRNA of one gene, 1 µM cisplatin or an equal volume of DMF was used to treat cells for 5 days. The cells were then incubated with 1:1000 Annexin V Alexa Fluor 488 to measure the percentage of apoptotic cells, 10 nM TMRE to examine the mitochondrial membrane potential, and 5 µM Draq5 to determine the nuclear morphology according to a previous study [16]. Finally, the cells were evaluated using the opera QEHS high-content screening system (PerkinElmer, USA). The images were collected and analyzed using the Acapella software (version 2.0; PerkinElmer, USA).

### Real-time quantitative PCR (RT-qPCR)

Total RNA was isolated with TRIZOL Reagent (Sigma-Aldrich) and reverse transcribed using a PrimeScript RT Master Mix kit (Takara, Japan) according to the manufacturer’s protocol. mRNA expression was determined by RT-PCR. The 2^−ΔΔCT^ method was used to quantify the mRNA expression. The primer sequences are listed in Supplementary Table S1.

### Western blot

The total protein lysates were prepared, subjected to SDS-PAGE, and transferred to PVDF membranes. To quantify the expression level of HNRNPU, membranes were probed with anti-HNRNPU antibody (catalog# 34,095, Cell Signaling Technology Inc., Danvers, MA). The loading control GAPDH was probed by anti-GAPDH antibody (Thermo Fisher Scientific Inc., Waltham, MA). Rabbit IgG HRP conjugates (Cell Signaling Technology Inc., Danvers, MA) were used as secondary antibody. To visualize the bands, ECL detection reagents were used (Thermo Fisher Scientific Inc., Waltham, MA). The protein band densitometry was measured using ImageJ software (NIH, MD).

### Cell proliferation assay

T24 cells were seeded in 96-well plates at 5000 cells per well. After treatment with 1 µM cisplatin or DMF for 1, 2, 3, 4, and 5 days, cell proliferation was examined using the Cell Counting kit-8 (CCK-8, Dojindo, Japan) following the manufacturer’s protocol. In brief, cells were collected at different time points and a 10 µL CCK-8 solution was added to each well. After incubation at 37 °C for 2 h, the light absorbance of each sample was measured at 450 nm using a microplate reader (Biotek).

### Flow cytometry

Cells were plated onto 6-cm dishes at 10^5^ cells per dish. After treatment with 1 µM cisplatin or DMF for 5 days, the cells were collected. For the apoptosis assay, cells were incubated with 10 μg/mL RNase A for 20 min, followed by treatment with 50 μg/mL propidium iodide (PI) solution for 20 min. Subsequently, the cells were stained with 10 μL Annexin V-FITC reaction reagent for 20 min. All the processes were performed at room temperature in the dark. For the cell cycle assay, the cells were fixed in 70% ethanol at -20 °C overnight after cisplatin treatment, followed by incubation with 50 μg/mL propidium iodide (PI) and 10 μg/mL RNase A at 37 °C for 30 min. Finally, the cells were analyzed using a flow cytometer (BD Biosciences), according to the manufacturer’s instructions.

### Cell apoptosis assay

After transfection, the cell culture supernatant of each experimental group was collected in a 5-mL centrifuge tube, washed once with D-Hanks. The cells were then trypsinized, the culture supernatant was terminated, and the cells were collected in the same 5-mL centrifuge tube. Cells were centrifuged at 1500 rpm for 5 min, the supernatant was discarded, and the cells were washed three times in PBS. The final density of the cell suspension was 1 × 10^6^—1 × 10^7^ cell/mL. A 1 × staining buffer was used to resuspend the cell pellet; 5 µL Annexin V-APC was used for staining, and cell pellets were transferred to a flow cytometer for analysis.

### Trans well assay

A total of 1.5 × 10^5^ cells were plated on the upper chamber of the transwell system, with 200 μL serum-free medium. Culture medium (800 μL) supplemented with 10% FBS was added to the lower chamber. After 12 h, the non-migrating cells were carefully removed using a cotton bud, and the migrated cells were fixed with 4% paraformaldehyde and incubated with 0.1% crystal violet. Cells were counted under a light microscope.

### ATAC-sequencing

After different treatments, the T24 cells were collected and incubated with 25 $\mu$ L transposase reaction solution (2 μL Tn5 transposase and 12.5 μL of TD buffer) at 37 °C for 1 h. Subsequently, the reaction was stopped by adding 25 μL EDTA solution. Next, the DNA fragments were enriched by PCR. The library was amplified using the following protocol: 72 °C for 5 min, 98 °C for 1 min, 15 cycles of 98 °C for 10 s, 63 °C for 30 s, and 72 °C for 1 min. Finally, the fragments were sequenced using a HiSeq2500 (Illumina, USA). Peak calling was analyzed using MACS2 [17, 18].

### ATAC-seq data analysis

Raw ATAC-seq fastq data were first cleaned using Cutadapt (3.4) to trim low-quality and adapter sequences. The cleaned sequence data was then aligned to the human hg19 genome reference using bowtie2 (2.4.4) in the ‘very-sensitive’ mode. Samtools (0.1.19) was then used to extract uniquely mapped alignments of high quality. To call ATAC-seq peaks, MACS2 (2.0.9) was used with the ‘nomodel’ option. The ‘shift’ and ‘extsize’ parameter of MACS2 was set as 100 and 200, respectively. The identified peaks were then annotated to the nearest genes by the homer (4.8). Pathway enrichment and motif analysis were performed using the online tools Enrichr and MEME-Suite, respectively.

### TCGA cohort analysis

The TCGA bladder cancer cohort was used for analysis. UALCAN, CbioPortal, and GEPIA were used for pan-cancer analysis and expression analysis of the HNRNPU gene [19, 20]. Promoter region methylation analysis was conducted using the UALCAN software. Survival and co-expression analyses were performed using the GEPIA web interface. GEPIA2021, along with the bioinformatics tools CIBERSORT, EPIC, and quanTIseq, was used to generate immune cell expression profiles of HNRNPU using the TCGA bladder cancer cohort.

### Microarray assay

The control cells (sgCtrl) and sgHNRNPU-transfected cells were treated with 1 µM cisplatin or DMF for 5 days. Total RNA from each group was isolated using the RNeasy Mini Kit (Qiagen), following the manufacturer’s protocol. Next, the samples were labeled and hybridized using the Affymetrix GeneChip Human Genome U133 Plus 2.0 Array, according to the manufacturer’s protocol. The data were analyzed using GeneSpring 12.6 software as previously described [21].

### Tumor xenograft model

T24 cells (1 × 10^7^ cells) with or without HNRNPU knockdown were suspended in 200 μL PBS and subcutaneously injected into each flank of 4–6-week-old BALB/c nu/nu female mice. The tumor volume was measured every 4 days. The mice were sacrificed after 28 days. Cisplatin was administered by tail injection at a dose of 2 mg/kg every other day. After 4 weeks, the mice were euthanized, the tumor was isolated, and the weight of the tumor was measured. Animal experiments were approved by the Xuzhou Medical University Animal Care and Use Committee.

### Statistical analysis

All experiments were independently repeated at least three times. The data were analyzed using GraphPad Prism 8.0 software, and shown as the mean ± standard deviation (SD). Two-tailed Student’s t-test was performed to compare the differences between two groups. One-way analysis of variance was performed to analyze the differences among multiple groups. Statistical significance was set at *P* < 0.05.

## Results

### Determination of cisplatin sensitivity in bladder cancer cell lines

**Fig. 1:**
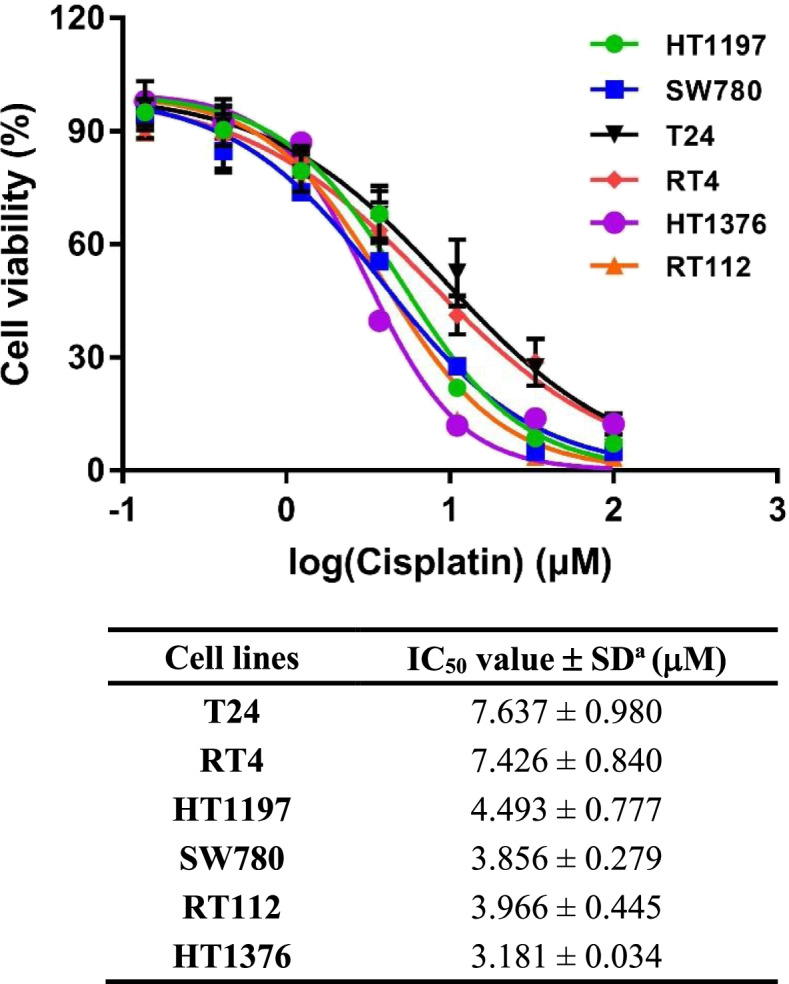
The cytotoxicity of cisplatin in 6 bladder cancer cell lines. Cell viability curves for T24, RT4, HT1197, SW780, RT112, and HT1376 cells. IC50 values are calculated using GraphPad software (Prism 7.0). Data are expressed as mean ± SD from a representative of three independent experiments

CCK-8 assay was performed to evaluate the cytotoxicity of cisplatin in six bladder cancer cell lines. As shown in Fig. 1, upon cisplatin treatment, T24 and RT4 had the highest IC_50_ values, that were 7.637 and 7.426 µM, respectively. HT1197, SW780, and RT112 shared similar IC_50_ values, while HT1376 was the most sensitive cell line to cisplatin. The cytotoxic profile suggested that T24 is the bladder cancer cell line which confers most resistant to cisplatin. Therefore, T24 cells were selected for further investigation.

### Generation and validation of the CRISPR system in BUC cells

To investigate genes that might be associated with cisplatin resistance in BUC, the GeCKO library containing 123,411 sgRNAs targeting 19,050 unique human genes was established and transfected into T24 cells. After puromycin selection, cisplatin (0.25 µM) was used to treat the cells for 7 or 14 days. Finally, the surviving cells were collected to examine the enriched sgRNAs using high-throughput sequencing. Our genome-wide CRISPR screening determined multiple enriched sgRNAs that might contribute to cisplatin response. Among them, 327 genes were significantly changed under cisplatin treatment, with 46 genes altered on day 7, and 284 genes altered on day 14, as compared to the day 0 group. As shown in (Supplementary Fig. 1A), GO analysis of these genes demonstrated that these genes were particularly enriched in the process of tissue development, organonitrogen compound metabolism, and ion transport. Meanwhile, we found that these genes were also related to cancer stem cells, that are closely associated with drug resistance (Supplementary Fig. 1B).

### HNRNPU depletion was associated with cisplatin sensitivity

**Fig. 2:**
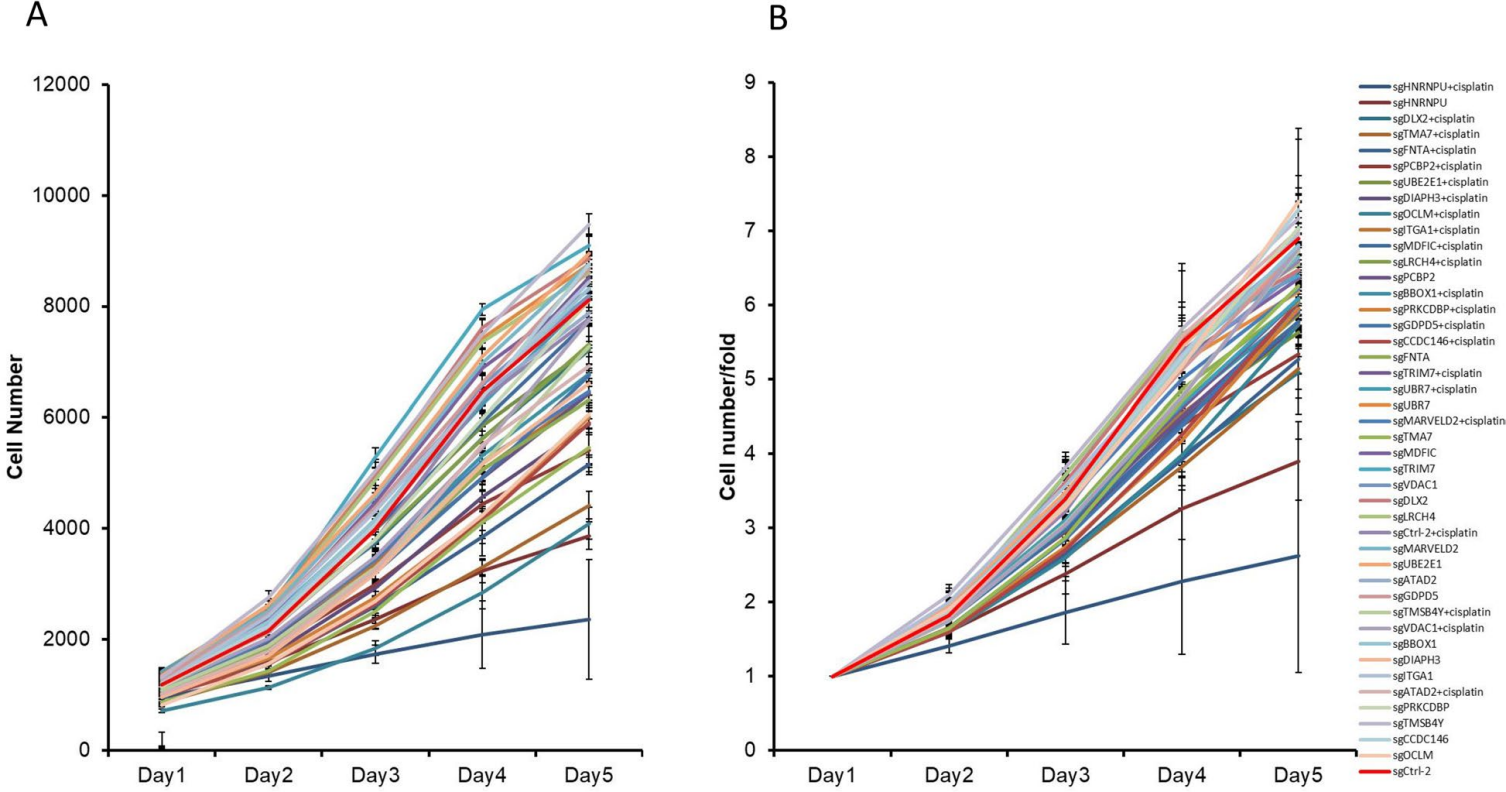
CRISPR-Cas9 screen to investigate mediators of cisplatin response in T24 cells. (**A**) Cell number count differences among different cisplatin/sgRNA combination treatments in the high-content screening assay. (**B**) Cell number foldchange of different cisplatin and sgRNA combination treatments compared to day 0. Error bar = SD

**Fig. 3:**
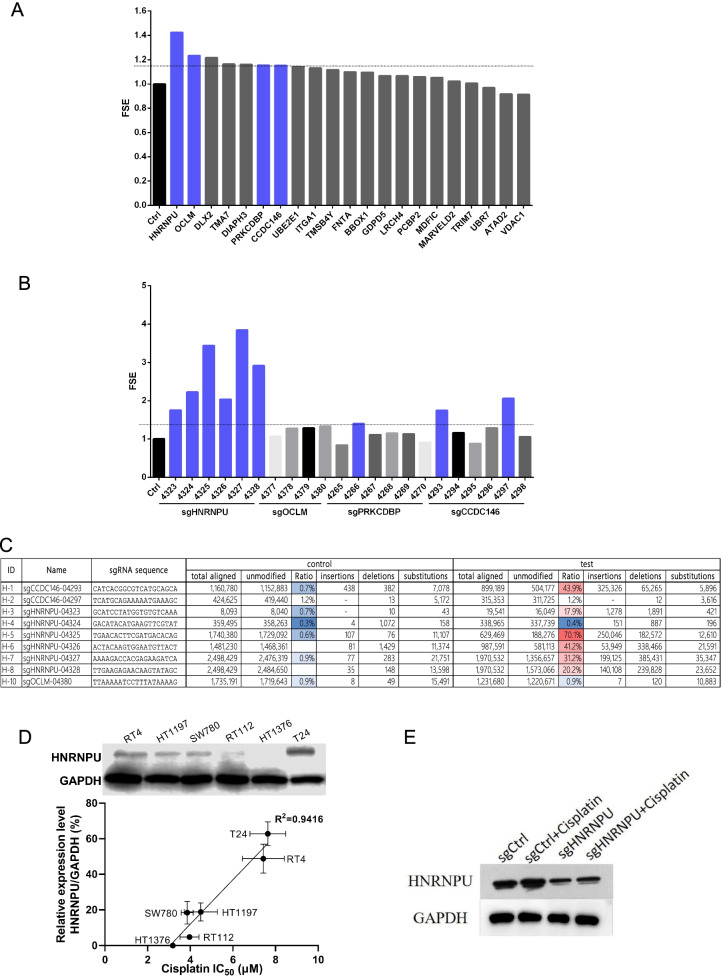
Loss of HNRNPU in T24 cells mediates cisplatin sensitivity in vitro*.* **(A)** Cells were transfected with six sgRNAs per gene, and the fold change of sensitivity enhancement (FSE) of the 21 top decreased genes in T24 cells from our screen were determined using HCS analysis. Results were normalized to the control group. FSE was calculated as the cell count fold change of ((NC + drug) /NC) / ((Treatment + drug) / Treatment), NC, negative control **(B)** sgRNA of four selected genes were transfected into cells, and the FSE of four selected genes, including HNRNPU, CCDC1, PRKCDBP and OCLM, were detected. **(C)** CRIPSR-based sgRNA editing efficiency of the top 9 sgRNA that targets the selected genes. (**D**) HNRNPU protein expression levels in T24, RT4, HT1197, SW780, RT112, and HT1376 cells. Simple linear regression of HNRNPU expression levels and cisplatin IC_50_ values in bladder cancer cells were calculated in GraphPad Prism 7.0 **(E)** Representative blot of HNRNPU in the cells after different treatments

To validate these results, 21 top decreased genes were selected. Six sgRNAs were designed for each gene, and the effects of these candidate targets were detected using HCS analysis. We first examined the cell count and fold change of cells on days 2–5 versus day 1. We found that treatment with many of the sgRNAs (HNRNPU, CCDC146, and more.) decreased the T24 cell proliferative rate compared to the sgCtrl treatment (Fig. 2A, B). The fold change in sensitivity enhancement (FSE) was increased in response to knockout of these genes. Among them, sgRNA treatments targeting HNRNPU, OCLM, PRKCDBP, and CCDC146 significantly decreased cisplatin resistance in T24 cells (FSE = 1.4) (Fig. 3A). We then tested the efficiency of each target sgRNA for four genes (HNRNPU, OCLM, PRKCDBP, and CCDC146) to improve the cisplatin response. The results showed that HNRNPU-04327 and HNRNPU-04325 transfection triggered a higher level of FSE than the other groups. For the CCDC146 gene, CCDC146-04,297 and CCDC146-04,293 were more effective in inhibiting cisplatin resistance. However, the targets for OCLM and PRKCDBP were similar to those of the control group (Fig. 3B). We also sequenced these sgRNA-targeted regions for FSE-related sgRNAs in T24 cells after the indicated treatments. Five of the six HNRNPU-targeted sgRNAs significantly edited the targeted genome region (Fig. 3C). Notably, sgHNRNPU-04325 had a 70.1% editing efficiency and had a significant impact on FSE (Fig. 3B). We then chose HNRNPU as a potential target to improve cisplatin sensitivity. As shown in Fig. 3D, a significant correlation was observed between HNRNPU protein expression levels and IC_50_ values of cisplatin. T24 cells, that had high HNRNPU expression level, were less sensitive to cisplatin. In contrast, HT1376, with no endogenous HNRNPU expression, was most sensitive to cisplatin. Therefore, we confirmed that HNRNPU could be a potential target to improve cisplatin sensitivity. Subsequently, the T24 cells were selected to establish an HNRNPU knockdown cell model. In addition, we tested the cytotoxicity of paclitaxel and doxorubicin, two chemotherapeutic drugs that are used for bladder cancer treatment (Supplementary Table 3**)**. Unlike cisplatin, no correlation was observed between the cytotoxic effect and HNRNPU expression level in the six bladder cancer cell lines, suggesting that HNRNPU mainly mediates the response of bladder cancer cells to cisplatin.

In order to validate the consequence of the genome editing effect of sgHNRNPU-04325, we performed Western blotting and showed that the protein level of HNRNPU was dramatically decreased compared to the control group. A similar trend was observed in sgRNA-transfected cells after cisplatin administration (Fig. 3E).

### Knockout of HNRNPU inhibited BUC progression by increasing cisplatin sensitivity

**Fig. 4:**
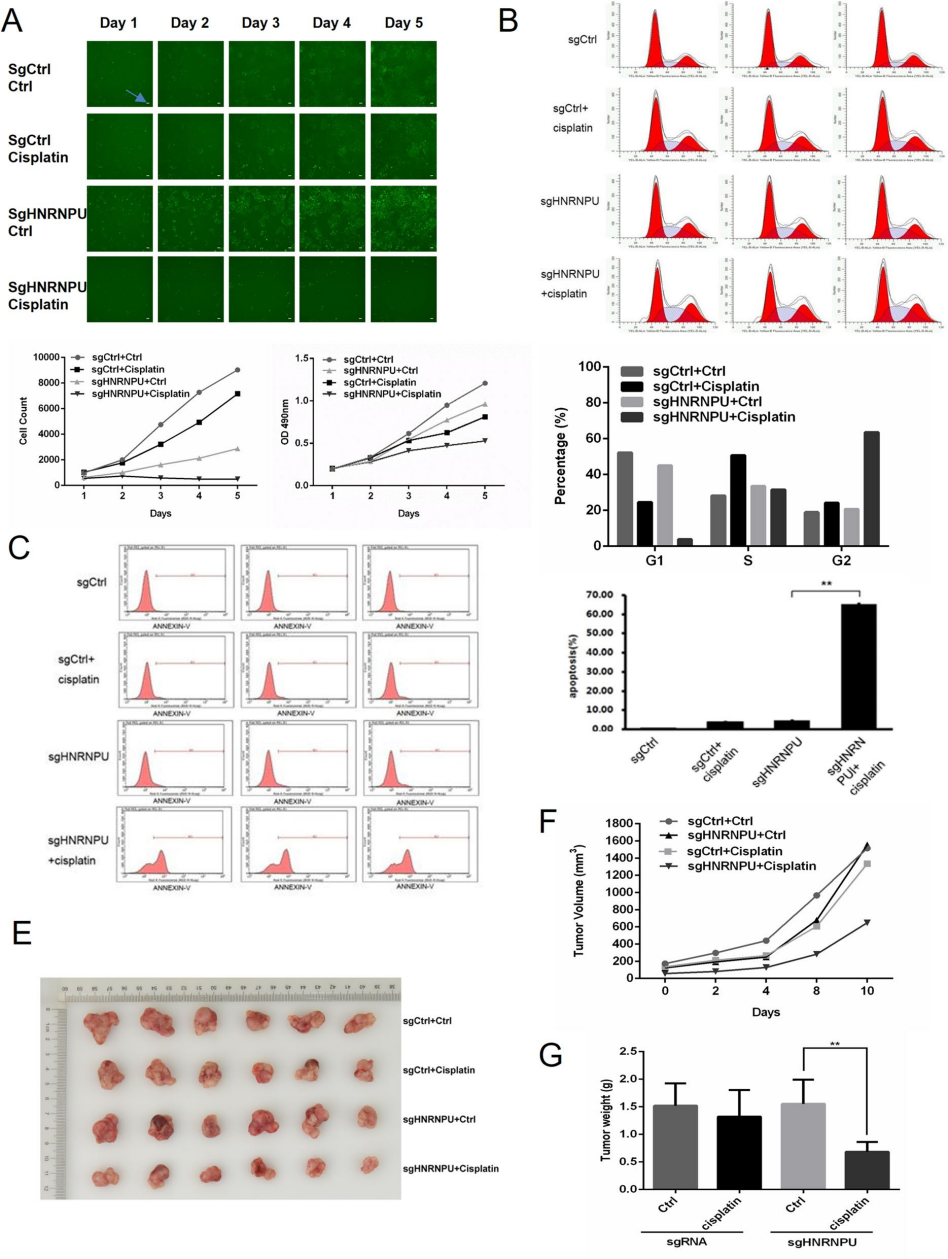
Knockout of HNRNPU regulated BUC progression and cisplatin sensitivity. **(A)** T24 cells were transfected with control sgRNA or sgHNRNPU prior to cisplatin treatment for 5 days, and cell viability was measured using CCK-8 assay. Arrows showed the error bars = 100 uM. **(B)** The cell cycle profiles of T24 cells with different treatments were detected by flow cytometry. Student’s t test was performed to detect the differences among treatments *: *p* < 0.05 **: *p* < 0.01**(C)** The apoptosis rate under the indicated treatments were examined by flow cytometry. **(D)** Cell migration of T24 cancer cells was determined by trans well assay. The data indicates mean ± SD from three independent experiments. **(E)** Photo of the isolated tumors with different treatments on day 28. **(F)** The tumor growth curve of different treatments, NC = negative control, KO = knockout of HNRNPU. **(G)** Weight of tumors in the indicated group

Next, we used HNRNPU-04325 sgRNAs for KO HNRNPU in T24 cells. We found that T24 cells were more sensitive to cisplatin in the sgHNRNPU transfection group than the sgCtrl + cisplatin group, with an overall cell count fold change of 7 versus 0.5 (day 5 compared to day 1). Notably, compared with the sgCtrl group, knockout of HNRNPU significantly decreased T24 cell viability, which was similar to the result of the sgCtrl + cisplatin group **(**Fig. 4A). HNRNPU inhibition leads to cell cycle arrest and cell apoptosis. As demonstrated in Fig. 4B, compared with the sgCtrl group, sgHNRNPU-treated T24 cells exhibited a dramatic increase in S-phase arrest. When cells were treated with cisplatin, this trend increased further. Consistently, the percentage of G1-phase was decreased. In addition, we observed a definite shift in apoptosis, with an approximately 60% increase in sgHNRNPU + cisplatin-treated cells compared to the other groups (Fig. 4C-D). These results indicate that HNRNPU downregulation increased cisplatin sensitivity.

We subsequently validated the effect of HNRNPU knockout on cisplatin sensitivity in vivo. As shown in Fig. 4E-G, cisplatin induced a slight decrease in tumor volume and tumor weight, while HNRNPU knockout significantly decreased the tumor size compared with cisplatin treatment alone, indicating that sgHNRNPU treatment enhanced the cisplatin sensitivity of the tumor.

### HNRNPU is highly expressed in almost all cancer types and is negatively correlated with survival outcome

**Fig. 5:**
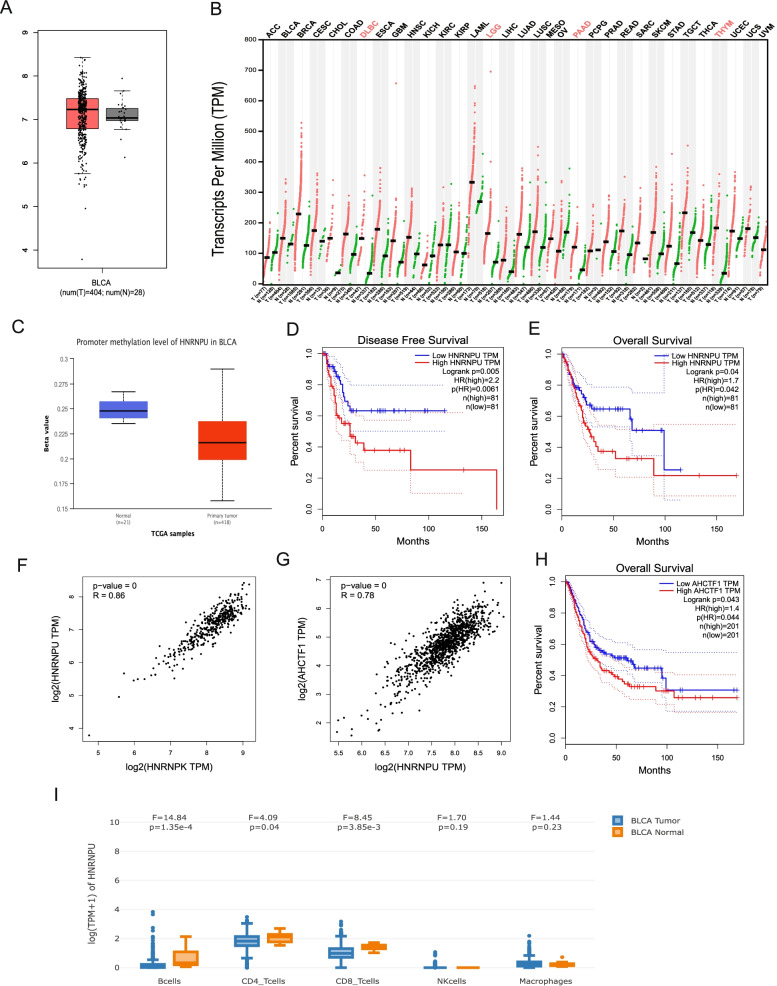
TCGA bladder cancer cohort analysis on HNRNPU expression and its impact on survival. (**A**) Expression profile of HNRNPU in bladder tumors versus control tissues in TCGA dataset. (**B**) Pan-cancer analysis of HNRNPU expression in multiple cancer types. (**C**) Promoter methylation level of HNRNPU in TCGA dataset (**D**). Disease free survival analysis of high HNRNPU patients versus low HNRNPU patients. (**E**) Overall survival analysis of high HNRNPU patients versus low HNRNPU patients (high = top 20%). (**F**) Co-expression correlation between HNRNPU and HNRNPK. (**G**) Co-expression correlation between HNRNPU and AHCTF1; R = Pearson’s r (**H**) Overall survival analysis between AHCTF1 high patients versus AHCTF1 low patients (high = top 50%). (**I**) Expression profile of HNRNPU in TCGA bladder cancer immune cell subsets. GEPIA2021 was used to generate the plot

We examined the overall expression level of HNRNPU in the TCGA bladder cancer cohort (T = 404) and found that HNRNPU was highly expressed in bladder tumors, compared to normal tissues (Fig. 5A). Interestingly, the expression level of HNRNPU was higher in tumor tissues than in normal tissues in almost all cancer types (Fig. 5B). We also observed a more hypomethylated promoter of the HNRNPU gene in bladder cancer tissues from the TCGA dataset, compared to the normal bladder tissues (Fig. 5C). We next divided TCGA bladder cancer patients into two groups, HNRNPU-high (Top 20%) and HNRNPU-low, and found that both the disease-free and overall survival rates of the HNRNPU-high patients were significantly lower than those in the HNRNPU-low group (Fig. 5D-E). These results indicate that high expression of HNRNPU in bladder cancer patients is negatively correlated with their clinical outcome.

We next explored whether genes highly correlated with HNRNPU could affect the survival of bladder cancer patients. Indeed, HNRNPK, an HNRNP family gene, was most significantly correlated with HNRNPU expression (Fig. 5F). However, different expression levels of HNRNPK do not affect the overall survival of patients with bladder cancer. Notably, AHCTF1, which was recently described as a MYC driver [22], is one of the most significantly correlated genes (Pearson's *r* = 0.78) (Fig. 5G). The overall survival rate of patients with high AHCTF1 was also significantly lower than that of patients with low AHCTF1 (Fig. 5H). Finally, we analyzed the expression of HNRNPU in the immune cells of bladder tumor tissues. Using the GEPIA2021 deconvolution tool for the TCGA bladder cancer cohort, we found that HNRNPU was significantly decreased in B cells, CD4^+^ T cells, and CD8^+^ T cells (Fig. 5I). Compared to its upregulation in tumor cells, the downregulation of HNRNPU in immune cells may also be associated with cisplatin resistance.

### HNRNPU KO sensitizes bladder cancer to cisplatin by rewiring the transcriptome and chromatin structure

**Fig. 6:**
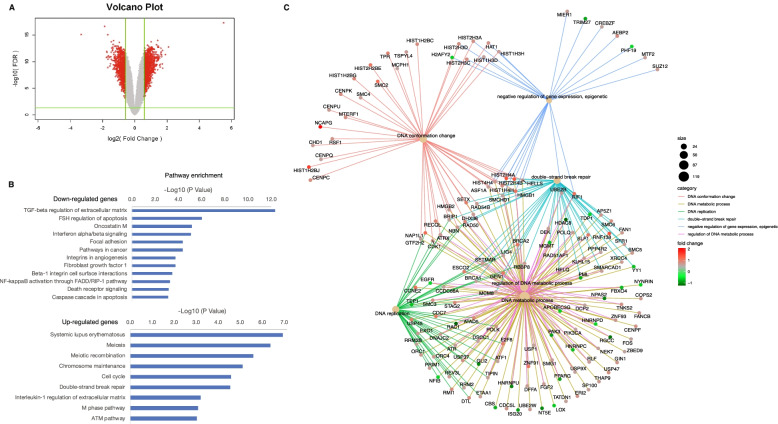
Differential expression analysis of HNRNPU-depleted cells. **(A)** Volcano plot showing the differentially expressed genes in HNRNPU-depleted cells. **(B)** Pathway analysis of the down-regulated genes and up-regulated genes. **(C)** CNEplot, which depicts the linkages of genes and biological concepts, reveals the top enriched pathways and genes involved in the indicated pathways

To investigate the mechanism by which HNRNPU knockout sensitizes BUC to cisplatin at the transcriptional level, we performed RNA-seq on both sgCtrl and sgHNRNPU groups, and identified 672 downregulated genes and 842 upregulated genes (Fig. 6A). The downregulated genes were enriched in several cancer-related pathways, such as “TGF-beta regulation of extracellular matrix,” “Oncostatin M,” “Focal adhesion” and “Pathways in cancer” (Fig. 6B). The upregulated genes were enriched in cell cycle-related pathways such as “Meiosis,” “Meiotic recombination,” “Cell cycle” as well as DNA repair pathways such as “double-strand break repair” and “ATM pathway.” These results demonstrate that HNRNPU knockout could rewire the transcriptome of bladder cancer cells, resulting in increased vulnerability to cisplatin treatment. Genes such as BRCA2, BRCA1, and RIF1, that are highly involved in DNA double-strand break repair pathways, were significantly upregulated in the HNRNPU knockout cells (Fig. 6C). Interestingly, AHCTF1 expression was significantly upregulated in the HNRNPU knockout group, indicating a potential interaction between AHCTF1 and HNRNPU (Supplementary Fig. 3, Fig. 5G).

**Fig. 7:**
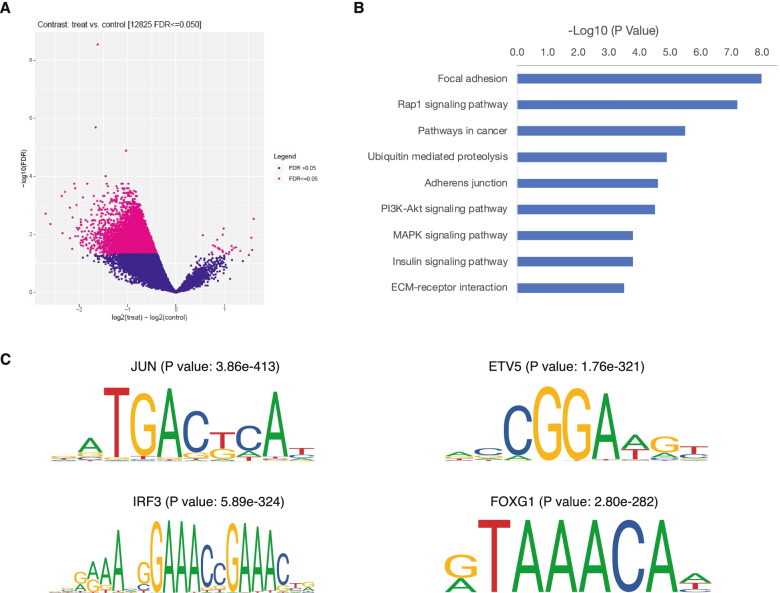
ATAC-seq analysis of HNRNPU-depleted cells. **(A)** Volcano plot showing the genomic regions with significant ATAC-seq signal alternations. Pathway analysis **(B)** and motif enrichment analysis **(C)** of the down-regulated regions in HNRNPU-depleted cells

A previous study reported that HNRNPU is an essential gene which regulates the interphase chromosome structure [23]. We assessed the alteration of interphase chromosome structure by ATAC-sequencing. Volcano plots showed the estimated log2(treat)-log2(control) *vs.* − log10 (false discovery rate, FDR) for each gene in the sgCtrl vs. the sgHNRNPU group. We identified 23 overrepresented genes and 12,833 downregulated genes in HNRNPU-deficient cells (FDR < 0.05) (Fig. 7A). Pathway analysis of the downregulated genes revealed that focal adhesion, the Rap1 signaling pathway, and pathways in cancers might be regulated under these conditions (Fig. 7B). Focal adhesion and pathways in cancers were also enriched in the downregulated genes, suggesting that the expression of these genes is regulated by chromatin openness. In contrast, the increased expression of genes in HNRNPU KO T24 cells was not accompanied by increased chromatin openness. It is speculated that transcription of these genes is likely to be regulated post-transcriptionally, possibly by regulating mRNA stability. To further investigate the transcription factors involved in regulating the downregulated genes, we performed motif analysis on the regions with decreased ATAC signal. Among the top enriched transcription factor motifs, we identified JUN, ETV5, and IRF3, that are well-known to be involved in cancer development. More interestingly, we also observed enrichment of the FOXG1 motif, which plays a crucial role in the oncogenesis of bladder cancer (Fig. 7C) [24, 25].

### HNRNPU regulate cisplatin resistance by regulating NF1.

We subsequently investigated the mechanism by which HNRNPU knockout enhances cisplatin sensitivity. We first selected the downstream gene of HNRNPU, from which the qPCR results showed the same trend as ATAC sequencing and microarray assay. Among the 25 downstream genes of HNRNPU verified by qRT-PCR (Supplementary Fig. 5), we chose nine genes that showed the same trend in qPCR and microarray to perform further analysis. Among the nine genes, only overexpression of Erb-B2 receptor tyrosine kinase 3 (ERBB3) and knockdown of neurofibromin 1 (NF1) exhibited a reverse regulation of HNRNPU knockout under cisplatin treatment, indicating that knockout of HNRNPU regulates the sensitivity of cisplatin treatment through ERBB3 or NF1 (Supplementary Fig. 5).

**Fig. 8:**
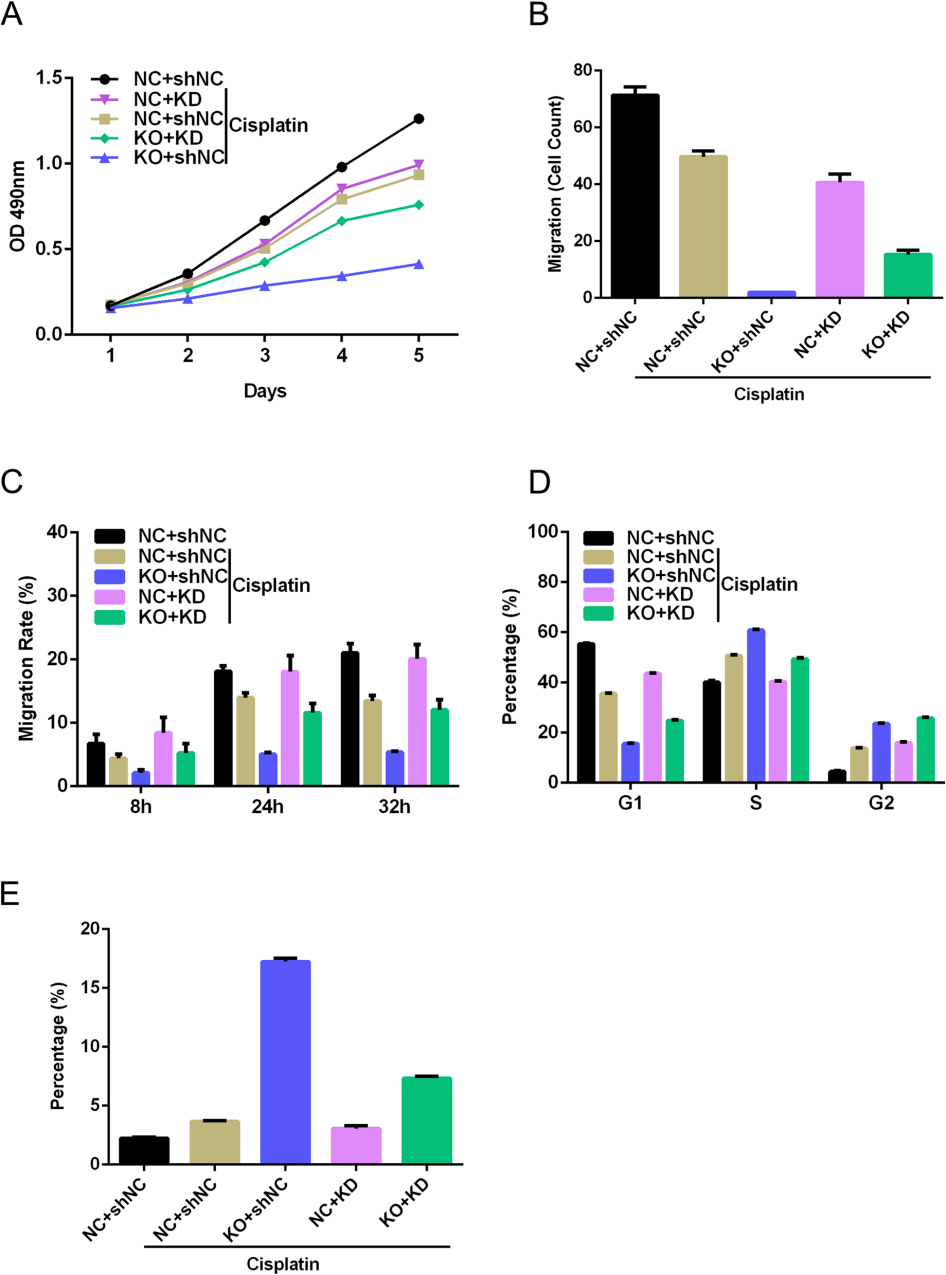
Knockout of HNRNPU regulated the BUC cell proliferation, apoptosis, and migratory through regulating NF1 expression**.** T24 cells were transfected with sgHNRNPU, with or without the NF1 knockdown, and then treated with or without cisplatin. **(A)** T24 cell viability was measured using CCK-8 assay. **(B)** Cell invasion of T24 cancer cells was determined by transwell assay. **(C)** Cell migration of T24 cancer cells was determined by wound healing assay. **(D)** The cell cycle of T24 cells with different treatments were detected by flow cytometry. **(E)** The apoptotic rate was examined by flow cytometry. The data indicate mean ± SD from three independent experiments. *p < 0.05, **p < 0.01, ***p < 0.001

We chose NF1 as the downstream target for validation, as NF1 has previously been associated with lower urinary tract dysfunction and resistance to EGFR inhibition in lung cancer [26, 27]. Using the NF1 knockdown lentivirus, we found that knockdown of NF1 abolished the effect of knockout of HNRNPU. Knockout of HNRNPU significantly increased the effect of cisplatin on the inhibition of cell proliferation, invasion, and migration, while knockdown of NF1 abrogated the effect of HNRNPU knockout, as indicated by the CCK-8 assay, transwell assay, and wound healing assay (Fig. 8A-C, Supplementary Fig. 6A-B). We further found that HNRNPU knockout enhanced cisplatin-induced cell cycle arrest in the S and G2 phases, which was reversed by NF1 knockdown (Fig. 8D, Supplementary Fig. 6C). Flow cytometry showed that knockout of HNRNPU increased cisplatin-induced apoptosis, while knockdown of NF1 reversed the effect of HNRNPU knockout on apoptosis (Fig. 8E, Supplementary Fig. 6D). These results suggest that knockout of HNRNPU increases cisplatin sensitivity by regulating NF1 related signaling pathway.

## Discussion

Cisplatin-based chemotherapy is a mainstay of BUC management. Previous studies have shown that DNA is a crucial target of cisplatin. Once activated, cisplatin can bind to the N7 reactive center, leading to DNA damage in cancer cells, inhibiting cell division, and inducing cell death [28, 29]. Unfortunately, cisplatin resistance is a common trait in cancer treatment. Moreover, some patients were not benefited from cisplatin chemotherapy because of the substantial side effects, such as hepatotoxicity and cardiotoxicity [28–30]. Hence, identifying the essential genes that associated with cisplatin response is important. Previous studies have shown that genome-wide CRISPR screening combined with NGS can be a useful tool to investigate potential genes or proteins to guide cisplatin treatment [15, 31]. In this study, we found that HNRNPU is involved in the response of bladder cancer to cisplatin, but not doxorubicin or paclitaxel in vitro. A low level of HNRNPU significantly improved cisplatin sensitivity compared to the other genes.

HNRNPU, which plays an essential role in cell survival against DNA damage, prompted DNA double-strand break repair, DNA-end resection, and ATR-dependent signaling via homologous recombination [32]. Down-regulation of HNRNPU expression led to inhibition of cell proliferation and was associated with good prognosis in cancer patients. In addition, Zhao et al. reported that this gene might promote cisplatin-induced apoptosis and increase drug sensitivity in lung squamous cell carcinoma [33]. HNRNPU is a possible marker for the diagnoses of multiple cancers, including pancreatic ductal adenocarcinoma, nasopharyngeal carcinoma, gastric cancer, and clear cell renal cell carcinoma [34–37]. Here, we identified that HNRNPU was significantly associated with cisplatin sensitivity using CRISPR screening. In a panel of bladder cancer cell lines, a significant correlation was found between HNRNPU protein level and the cytotoxicity of cisplatin. Downregulation of HNRNPU is profoundly related to cisplatin chemosensitivity. Furthermore, we demonstrated that knockout of HNRNPU in combination with cisplatin treatment induced apoptosis, S-phase arrest, and inhibited cell migration in a cisplatin-resistant bladder cancer cell line. By analysis of the TCGA bladder cancer cohort, we also found that high HNRNPU is negatively correlated with patient survival, indicating its negative effect on cancer treatment. In a validation analysis of another cohort of bladder cancer (PMID: 23,897,969), the association between high HNRNPU and poor survival outcome was also observed, though the statistic result is not significant, which may be due to the limited case number (Supplementary Fig. 7).

In order to demonstrate the mechanism by which HNRNPU is linked to cisplatin sensitivity, we performed ATAC-sequencing and microarray assays on the HNRNPU KO and control cells. The resulting data showed that the interphase chromosome structure of the majority of genes was downregulated when HNRNPU expression was inhibited in T24 cells. Among them, most genes showed gene expression, transcription, and focal adhesion. Interestingly, from the microarray data of HNRNPU-depleted T24 cells after cisplatin treatment, we observed that cisplatin significantly modified the response to DNA damage, and hence influenced cell cycle arrest and apoptosis. Our results demonstrated that HNRNPU is involved in sensitization to cisplatin by regulating the ATM and DNA double-strand break repair pathways.

We further investigated the downstream genes regulated by HNRNPU, which mediates the chemosensitivity of HNRNPU. CCK-8 assay with the expression profile assays revealed that only knockdown of NF1 or overexpression of ERBB3 could reverse the effect of HNRNPU knockout on cisplatin sensitivity. NF1 is a tumor suppressor that regulates RAS via modulating GTPase activity [38]. Mymryk et al. reported that cisplatin inhibits chromatin remodeling and transcription factor binding of NF1 in mice [39]. In melanoma and lung cancer, loss of NF1 conferred chemoresistance to cancer cells through inhibition of kinases and the RAS signaling pathways [26, 40]. In the present study, we found that HNRNPU knockout induced upregulation of NF1, and knockdown of NF1 reversed the effect of HNRNPU knockout on enhancing chemosensitivity, indicating that the effect of HNRNPU was mediated by regulating NF1 expression. However, how HNRNPU determines NF1 activity is not fully understood. The regulatory axis of HNRNPU and NF1 warrants further study. Moreover, the role of ERBB3 was not verified because of the failed overexpression of ERBB3 protein in cells, and the relationship between ERBB3 and HNRNPU should be explored in the future.

## Conclusions

In summary, our findings demonstrated that CRISPR screening is a useful tool for identifying potential targets to enhance cisplatin sensitivity. Our study identified several novel genes associated with cisplatin sensitivity via in vitro screening and identified the top candidate gene HNRNPU. Knockout of HNRNPU inhibits cell proliferation and migration. Moreover, our work provided evidence that the loss of HNRNPU enhanced cisplatin sensitization by regulating the interphase chromosome structure of genes in DNA damage repair pathways. Further studies showed that HNRNPU regulated chemosensitivity by regulating NF1. Our study provides a novel insight that inhibiting HNRNPU could be a promising strategy to improve cisplatin sensitivity in patients with BUC.

## Supplementary Information

**Additional file 1.** 

## Data Availability

For all data requests, please contact the corresponding authors.

## Abbreviations

CRISPR: Clustered regularly interspaced short palindrome repeat-associated nuclease
HNRNPU: Heterogeneous nuclear ribonucleoprotein U
BUC: Bladder urothelial carcinoma
TCGA: The Cancer Genome Atlas
NF1: Neurofibromin 1
sgCtrl: Control sgRNA
MOI: Multiplicity of infection
DMF: Dimethylformamide
GO: Gene ontology
PI: Propidium iodide
SD: Standard deviation
ERBB3: Tyrosine kinase receptor3
