# Supplementary material for: Targeting HNRNPU to overcome cisplatin resistance in bladder cancer
Source: Mol Cancer. 2022 Feb 7;21:37. doi: 10.1186/s12943-022-01517-9 (PMC8819945; doi:10.1186/s12943-022-01517-9)
Supplement: Supplementary file 1 — Additional file 1. [file 12943_2022_1517_MOESM1_ESM.pdf]

**Supplementary Materials**

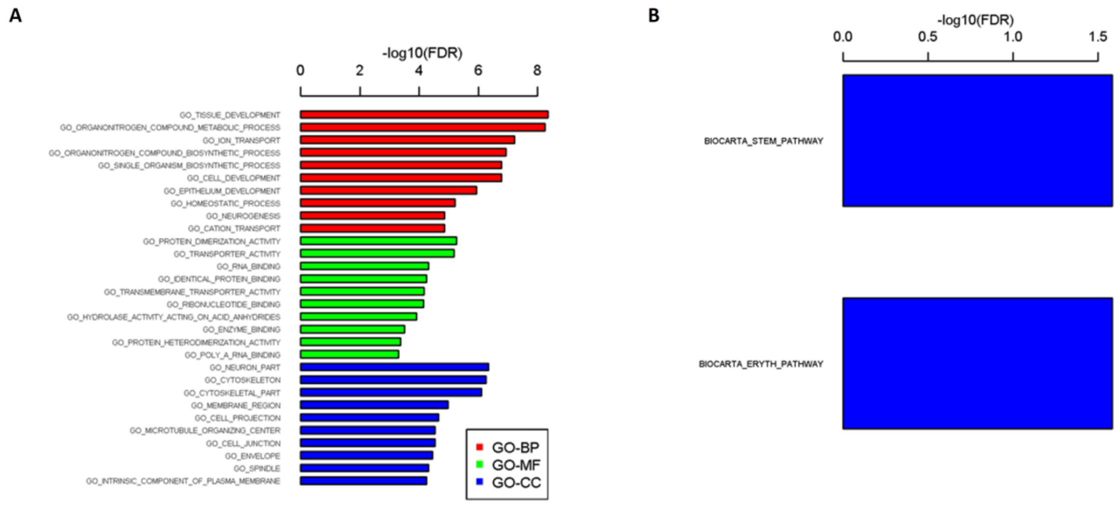


**Supplementary Figure 1.** Pathways related to the CRISPR-Cas9 screen investigating mediators of cisplatin response in T24 cells. (A) Top GO analysis and (B) pathway analysis of the 327 regulated genes in T24 cells after CRISPR-cas9 genome editing.

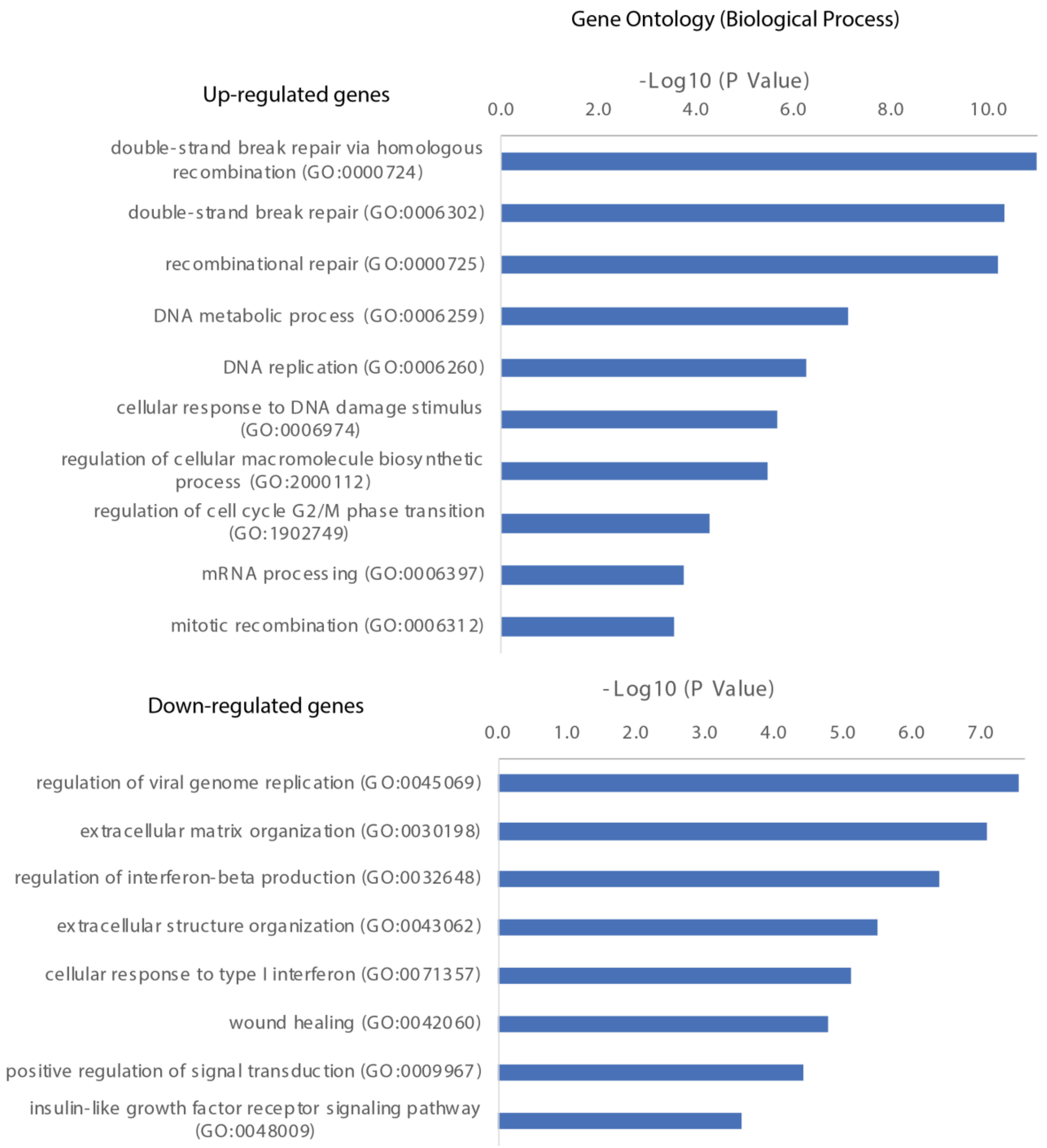


**Supplementary Figure 2**. Gene ontology pathway enrichment of the microarray data. Enrichment of both upregulated and downregulated DEG (differentially expressed genes) is shown.

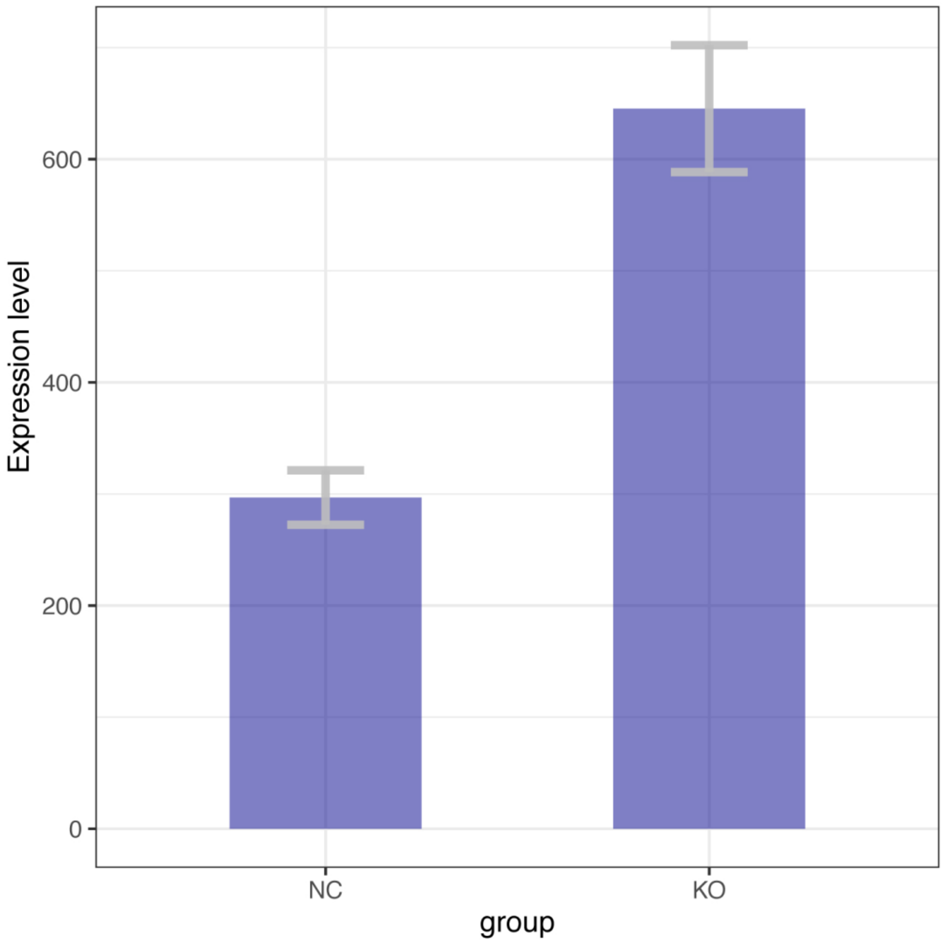


**Supplementary Figure 3**. AHCTF1 expression profile from HNRNPU-KO microarray dataset.

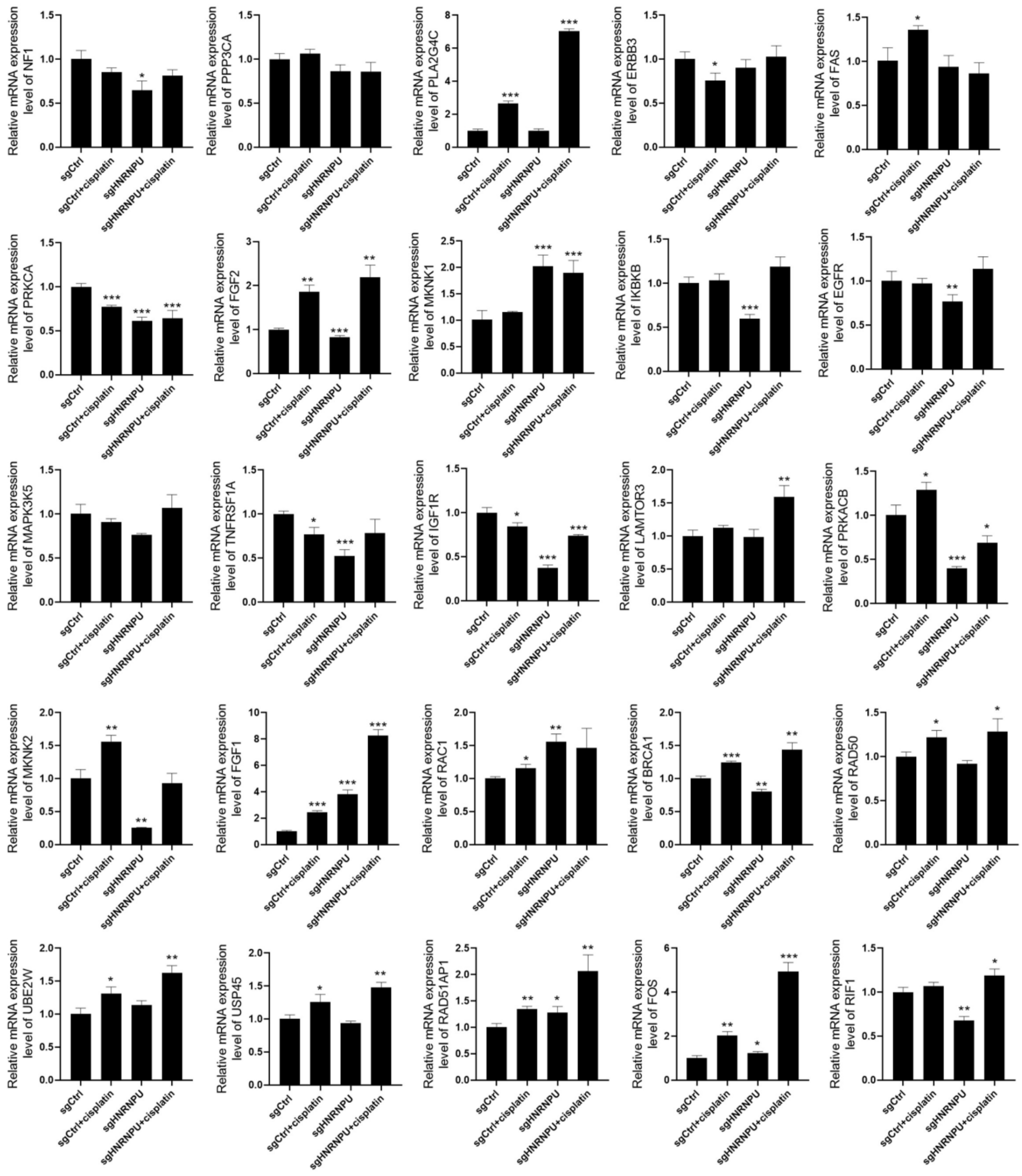


**Supplementary Figure 4.** Validation of the MAPK pathway and DNA double-strand break repair gene expressed in the HNRNPU-depleted cells after cisplatin treatment. T24 cells were transfected with sgHNRNPU and then treated with cisplatin for 5 days. The qRT-PCR assay was used to detect the mRNA expression of genes. The data indicate mean ± SD from three independent experiments. The data indicate mean ± SD from three independent experiments. *p<0.05, **p<0.01, ***p<0.001 vs. the sgCtrl group.

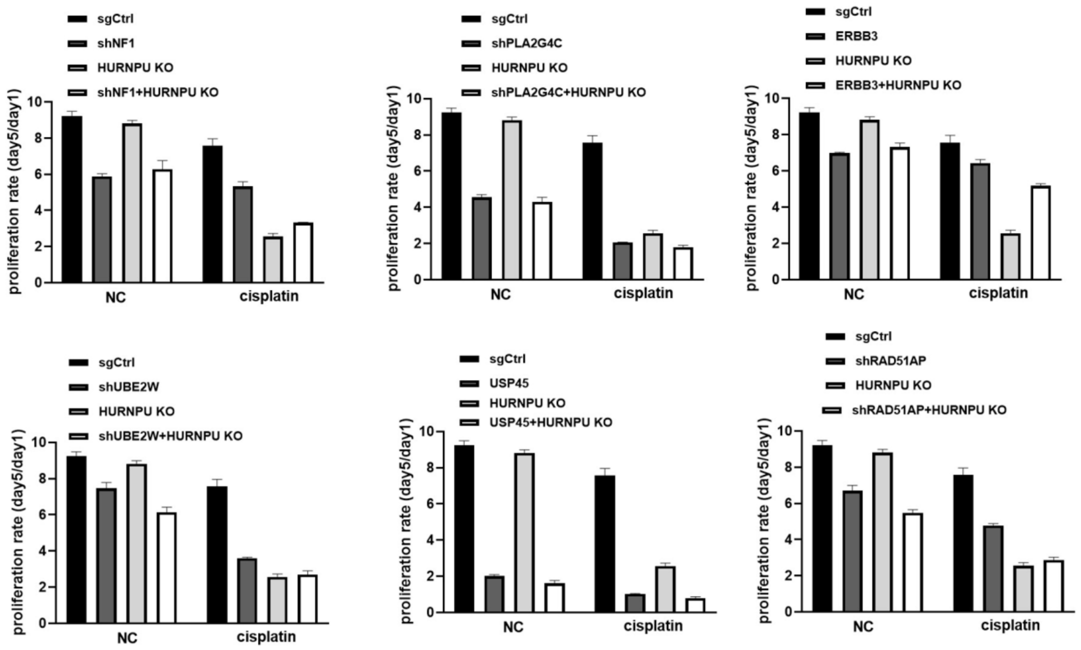


**Supplementary Figure 5.** Screening of HNRNPU downstream genes. T24 cells were transfected with sgHNRNPU, with or without the downstream gene knockdown or overexpression, and then treated with or without cisplatin for 5 days. The CCK-8 assay was used to detect the cell proliferation in the indicated group.

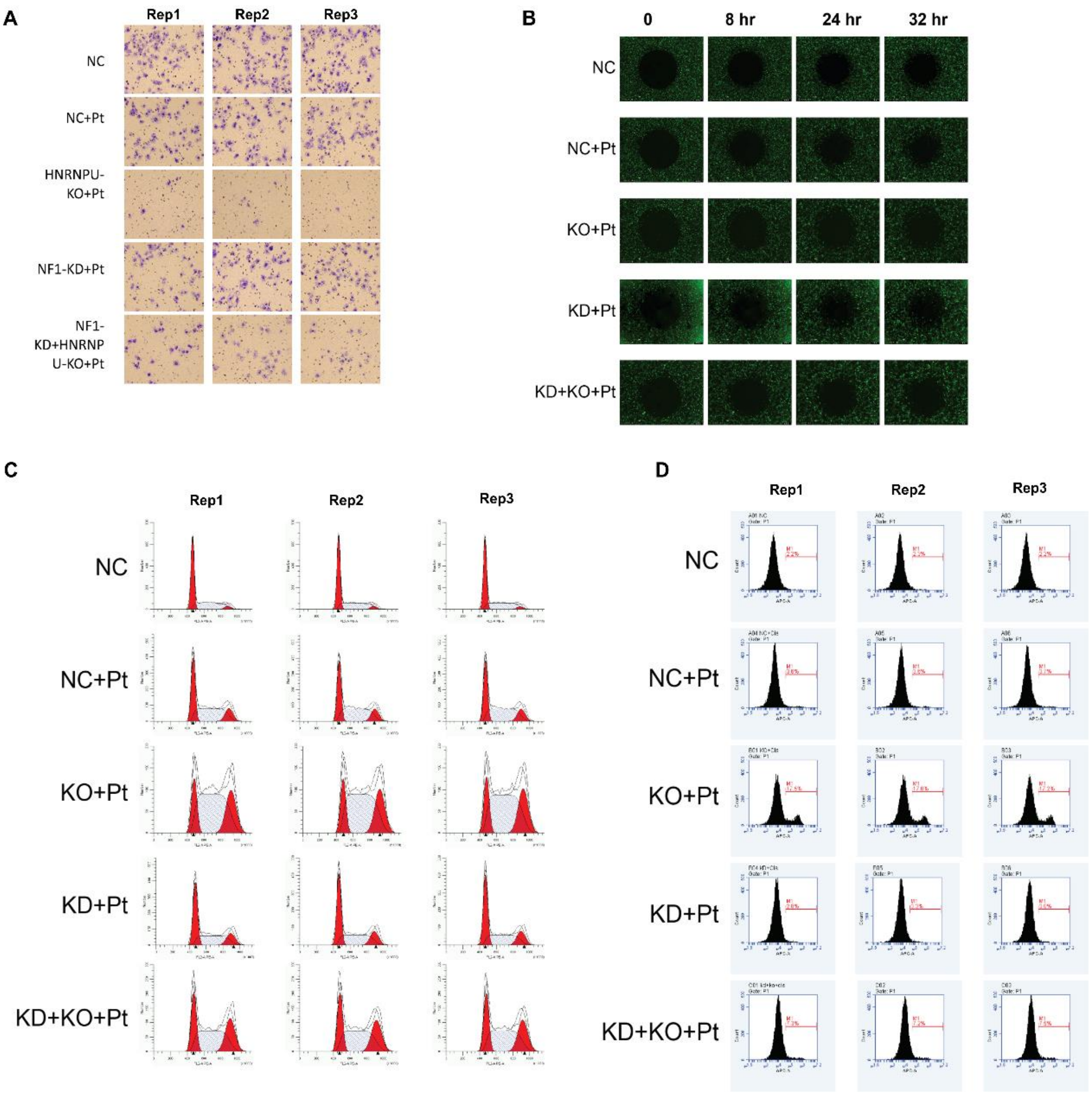


**Supplementary Figure 6.** A. Cell invasion assay results under different NF1, HNRNPU KO or combination treatments. Rep = replication. **B.** Cell migration assay results under different NF1, HNRNPU KO or combination treatments. Hr= hour. **C.** Cell cycle arrest results under different NF1, HNRNPU KO or combination treatments. Rep = replication. **D.** Cell apoptosis results under different NF1, HNRNPU KO or combination treatments. Rep = replication.

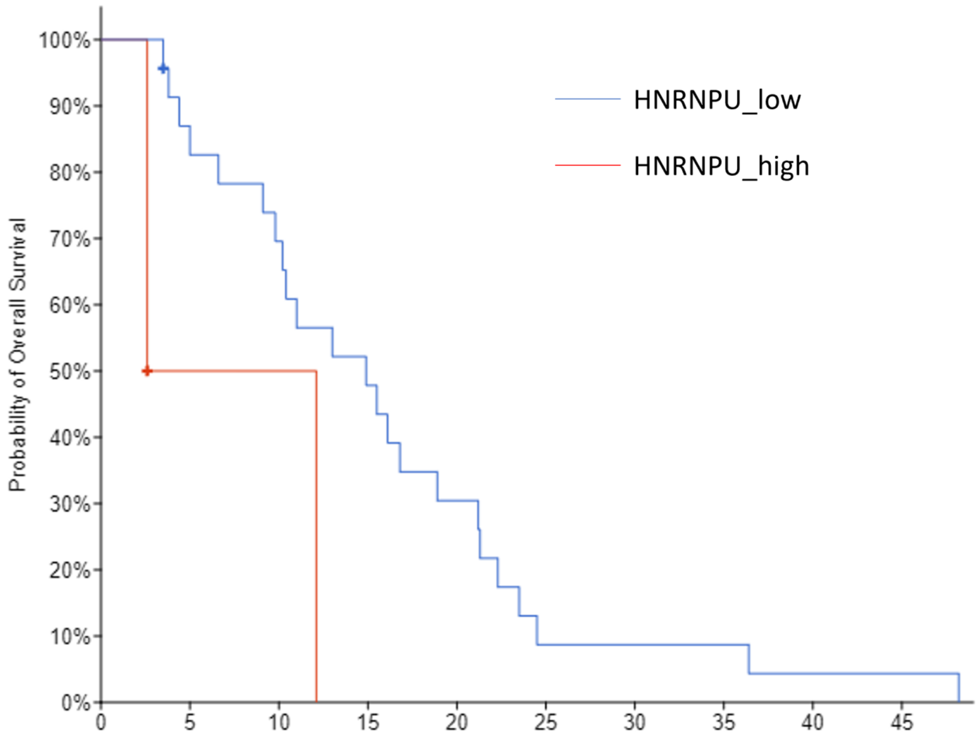


**Supplementary Figure 7. Survival outcome of patients with low HNRNPU (blue line) and high HNRNPU expression (red line).** Data was from Iyer et al., 2013, total case number = 59.

**Supplementary Table S1. Primers of genes for RT-PCR.**

| Gene | Sequences |
|---|---|
| NF1 | F 5'-TCCATCCCCTTTATCCCAGCC-3'<br>R 5'-CCAGTTAGGAGGGTCACGGA-3' |
| PPP3CA | F 5'-GCCGCGGACCTTCTAGGATA-3'<br>R 5'-TGACGCCCCCGTCAATAAAA-3' |
| PLA2G4C | F 5'-CACGTGATCCCACGGATGAA-3'<br>R 5'-TGCACCCAATCCCTGGTACT-3' |
| ERBB3 | F 5'-CTTGCCTCGATGTCCTAGCC-3'<br>R 5'- CTGAGACCGTGCCCATACC-3' |
| FAS | F 5'-CAAGAATTGCCAGGCGAACAA-3'<br>R 5'-TTGCATCCCAAACAGAGCCA-3' |
| PRKCA | F 5'-AAGAACGTGCACGAGGTGAA-3'<br>R 5'-CCCGCCGGTTCCAAGTTATC-3' |
| FGF2 | F 5'-TCCATTTCGTGGGTTCTCGC-3'<br>R 5'-AAATTGGCACACCCCAAAGC-3' |
| FOS | F 5'-GCGGTAGGTACTCTGTGGGT-3'<br>R 5'-AGGTTGGCAATCTCGGTCTG-3' |
| MKNK1 | F 5'-GTTCTCGGAGGAGCGATCTG-3'<br>R 5'-GAAAAGCGTCCCCTTAGGCT-3' |
| IKBKB | F 5'-GGTCACCTTCCCTGACAACG-3'<br>R 5'-GTCCCAAGATGACCAAAGCCA-3' |
| EGFR | F 5'-CCAGTATTGATCGGGAGAGCC-3'<br>R 5'-CGCAGCTGATCTCAAGGAAA-3' |
| MAPK3K5 | F 5'-CTTTGGAGAAACCACCGTGC-3'<br>R 5'-TAAATGCTTCCCGCCCAGAA-3' |
| TNFRSF1A | F 5'-CTATGCCCGAGTCTCAACCC-3'<br>R 5'-CCCTTTGTCCCTGGTCTCAC-3' |
| IGF1R | F 5'-GTTTTTGGAGGGGGAGCGAA-3'<br>R 5'-GCCCCTCGGAGGAAAAGTT-3' |
| LAMTOR3 | F 5'-GGCTTGAGGAGGAACCTGTC-3'<br>R 5'-GGCCTCAAAACCAGGCAAAG-3' |
| PRKACB | F 5'-TTGTCCCAGACTGTGGAGTG-3'<br>R 5'-GCCTTCAACTCACCGCTCTC-3' |
| MKNK2 | F 5'-TTCCACCGTTCGTTCAAGGT-3'<br>R 5'-GAGCCGATCTTAGGGGCG-3' |
| FGF1 | F 5'-CTGCAGCTGTCCTGGTAGAA-3'<br>R 5'-GCCAAGCAGAAAGTCTGTAGC-3' |
| RAC1 | F 5'-AGCAAGCGCTCTTGGAGATT-3'<br>R 5'-TATGATCGACTTGACCGCCG-3' |
| BRCA1 | F 5'-TCCCATCTGGTAAGTCAGCAC-3'<br>R 5'-CCGGACCACAGGATTTGTGT-3' |
| RIF1 | F 3'-AGTTTTGCCAGTTGACTCAAAAGC-3'<br>R 5'-TGCCCCTAGGCAACTTTAGG-3' |
| RAD50 | F 5'-ACGATCCCAAGGTAATGGTGC-3'<br>R 5'-GTGCTAAAACTGTGTGGGGC-3' |
| UBE2W | F 5'-TGTCAGTGCGTCTCAACCAT-3'<br>R 5'-GTAGAGCGTCCCCAGCATTT-3' |
| USP45 | F 5'-GGGCTTTTTGCATAGCCTGTT-3'<br>R 5'-CCATTTGAGGCAAAAGGGCA-3' |
| RAD51AP1 | F 5'-AACTTGGGCAGAGTCATAGGTC-3'<br>R 5'-GTGCTGGGTCCTGTCAAAAC-3' |

**Supplementary Table S2.** Student's t-test results of Figure 4G with indicated comparison.

| T-TEST_tumor weight | P VALUE |
|---|---|
| NC VS NC+Cisplatin | 0.496145642 |
| NC VS KO | 0.87067181 |
| NC VS KO+Cisplatin | 0.00069077 |
| NC+Cisplatin VS KO | 0.426861325 |
| NC+Cisplatin VS KO+Cisplatin | 0.010703857 |
| KO VS KO+Cisplatin | 0.000833118 |

**Supplementary Table S3.** Drug sensitivity of bladder cancer cell lines to chemotherapeutic drugs.

| | $IC_{50}$ values ± SD [a] (μM) | | |
|---|---|---|---|
| Treatment | Cisplatin | Doxorubicin | Paclitaxel |
| T24 | 7.637±0.980 | 0.341±0.043 | 0.066±0.013 |
| RT4 | 7.426±0.840 | 0.193±0.002 | 0.337±0.110 |
| HT1197 | 4.493±0.777 | 0.266±0.010 | 0.301±0.051 |
| SW780 | 3.856±0.279 | 0.135±0.009 | 0.029±0.002 |
| RT112 | 3.966±0.445 | 0.094±0.007 | <1 nM |
| HT1376 | 3.181±0.034 | 0.300±0.115 | 0.258±0.086 |

[a] $IC_{50}$ values are represented as the mean ± SD of at least three independent experiments.
